# Are the Mesothelial-to-Mesenchymal Transition, Sclerotic Peritonitis Syndromes, and Encapsulating Peritoneal Sclerosis Part of the Same Process?

**DOI:** 10.1155/2013/263285

**Published:** 2013-02-10

**Authors:** Jesús Loureiro, Guadalupe Gónzalez-Mateo, José Jimenez-Heffernan, Rafael Selgas, Manuel López-Cabrera, Abelardo Aguilera Peralta

**Affiliations:** ^1^Centro de Biología Molecular Severo Ochoa, CSIC-UAM, Campus de Cantoblanco, Calle de Nicolás Cabrera 1, 28049 Madrid, Spain; ^2^Servicio de Nefrología, Hospital Universitario La Paz, Instituto de Investigación Sanitaria La Paz (IdiPAZ), Paseo de la Castellana, 261, 28046 Madrid, Spain; ^3^Servicio de Anatomía Patológica, Hospital Universitario de la Princesa, Instituto de Investigación Sanitaria Princesa (IP), Calle de Diego de León 62, 28006 Madrid, Spain; ^4^Unidad de Biología Molecular and Servicio de Nefrología, Hospital Universitario de la Princesa, Instituto de Investigación Sanitaria Princesa (IP), Calle de Diego de León 62, 28006 Madrid, Spain

## Abstract

Mesothelial-to-mesenchymal transition (MMT) is an autoregulated physiological process of tissue repair that in uncontrolled conditions, such as peritoneal dialysis (PD), can lead to peritoneal fibrosis. The maximum expression of sclerotic peritoneal syndromes (SPS) is the encapsulating peritoneal sclerosis (EPS) for which no specific treatment exists. The SPS includes a wide range of peritoneal fibrosis that appears progressively and is considered as a reversible process, while EPS does not. EPS is a serious complication of PD characterized by a progressive intra-abdominal inflammatory process that results in bridles and severe fibrous tissue formation which cover and constrict the viscera. Recent studies show that transdifferentiated mesothelial cells isolated from the PD effluent correlate very well with the clinical events such as the number of hemoperitoneum and peritonitis, as well as with PD function (lower ultrafiltration and high Cr-MTC). In addition, in peritoneal biopsies from PD patients, the MMT correlates very well with anatomical changes (fibrosis and angiogenesis). However, the pathway to reach EPS from SPS has not been fully and completely established. Herein, we present important evidence pointing to the MMT that is present in the initial peritoneal fibrosis stages and it is perpetual over time, with at least theoretical possibility that MMT initiated the fibrosing process to reach EPS.

## 1. Introduction

Peritoneal dialysis (PD) is a form of renal replacement therapy that uses the peritoneal membrane (PM) as semipermeable barrier for the exchange of toxic substances and water. This technique has increased during the last years, in parallel to its complications. Currently, prolonged survival on PD has been reached due to technological advances, prevention, and early diagnosis of uremic complications. The basic objective of DP is the long-term preservation of the PM function. The PM is lined by a monolayer of MCs that have characteristics of epithelial cells and act as a permeability barrier across which ultrafiltration and diffusion take place. The long-term exposure to hyperosmotic, hyperglycaemic, and low pH of dialysis solutions and repeated episodes of peritonitis or hemoperitoneum cause injury of the peritoneum, which progressively becomes denuded of MCs and undergoes fibrosis and neovascularization [1]. Such structural alterations are considered the major cause of ultrafiltration failure [1, 2]. In this context, it has been proposed that local production of vascular endothelial growth factor (VEGF), a potent proangiogenic cytokine, during PD plays a central role in processes leading to peritoneal angiogenesis and functional decline [2–5]. Recently, it has recognized the role of transdifferentiation of mesothelial cells (MMT) in peritoneal fibrosis, angiogenesis, lymphangiogenesis, and PM failure. The process is governed by the transforming growth factor-*β* (TGF-*β*) and the representative cell form is the myofibroblast. TGF-*β* synthesis may be stimulated by glucose, and infections, via peritoneal leucocyte-derived factors and it is considered the master molecule of tissue fibrosis [6, 7]. The maximum expression of peritoneal fibrosis or sclerotic peritoneal syndromes (SPS) induced by PD fluids is the encapsulating peritoneal sclerosis (EPS) which is a serious complication of PD [8, 9]. The SPS is traditionally considered as a reversible process, while EPS is not. Emerging evidences have indicated that MMT is persistently present in initial and end stages of peritoneal fibrosis [10–12]. Moreover, its significant blockade decreases the peritoneal damage induced by PD fluids, including fibrosis and angiogenesis [13, 14]. These findings suggest that there is a chain between MMT and SPS. But the jump from SPS to EPS has not yet been fully established. Here we review current data regarding a possible connection between MMT, SPS, and EPS, considering the MMT as a new process in PD presumably involved in the deterioration phases of PM.

## 2. The Peritoneal Membrane Fibrosis in PD 

Peritoneal fibrosis (or sclerosis) is a term that comprises a wide spectrum of peritoneal structural alterations, ranging from mild inflammation to severe sclerosing peritonitis and its most complicated manifestation, encapsulating peritonitis sclerosis (EPS) [8, 9, 15]. Simple sclerosis (SS), an intermediate stage of peritoneal fibrosis, is the most common peritoneal lesion found in the patients after few months on PD and could represent the initial phase of sclerosing peritonitis syndrome (SPS). Rubin et al. [16] described a normal thickness of the peritoneum of 20 *μ*m, which after a few months on PD could reach up to 40 *μ*m (SS). The SPS is a progressive sclerosis that is characterized by a dramatic thickening of the peritoneum (up to 4000 *μ*m) and is accompanied by inflammatory infiltrates, calcification, neovascularization, and dilatation of blood and lymphatic vessels, being the most thickening commonly used pathological criterion for differential diagnosis [17–19]. In some instances, granulated tissue is observed to immerse in exudates containing fibrin and giant cells, probably reflecting chronic inflammation. Peritoneal fibrosis consists in the accumulation of ECM proteins (collagen I, III, V, VI, fibronectin, tenascin) in the interstitium, with augmented number of fibroblasts, some of them with myofibroblastic features, and mononuclear cell infiltration. In the basement membrane there is usually accumulation of collagen IV and laminin and proteoglycans, and polysaccharides and glycoproteins are also present extracellularly [8, 9, 15]. In 2003, our group discovered that MCs undergoes a process of transdifferentiation that is so-called epithelial to mesenchymal transition (EMT) or mesothelial to mesenchymal transition (MMT) by the negative effects of PD liquids [10]. 

### 2.1. Mesothelial-to-Mesenchymal Transition (MMT)

MMT is a complex and generally reversible process that starts with the disruption of intercellular junctions and loss of apical-basolateral polarity, typical of epithelial cells, which are then transformed into fibroblast-like cells with increased migratory, invasive, and fibrogenic features. The objective of this process is to repair tissue wounds by promoting the recovery of ancestor capabilities of epithelial cells. Cell migration, production of extracellular matrix, and induction of neoangiogenesis are the main activities [20]. The process is conducted by the transforming growth factor-*β* (TGF-*β*). 

### 2.2. TGF-*β* Is the Master Molecule in MMT and Peritoneal Fibrosis Pathway

TGF-*β* is a growth factor implicated as the causal agent in fibrosis of different tissues and organs [7]. This exists in tissues, generally in a latent and inactive form, bound to the latency-associated peptide (LAP), and it is activated through proteolytic cleavage by thrombospondin, plasmin, cathepsin D, furin, and glycosidases when exposed to PD solutions [21]. Its synthesis may be stimulated by glucose, acid pH, and infections, via peritoneal leucocyte-derived factors and its overexpression has been correlated to worse PD outcomes [22–25].

Four different intracellular signal pathways are triggered upon engagement of TGF-*β* to its receptors, being the most important as the Smads cascade [26–30]. Clinically, the factors involved in the stimulation of TGF-*β* and the initiation of SPS include the following. 

(1) Peritonitis is one of the most commonly invoked pathogenic factors for SPS [8, 9]. Some etiological agents have been identified including the bacteria *Staphylococcus aureus, Pseudomona* sp., and *Haemophilus influenza*. These pathogens promote conversion of fibrinogen by coagulase to a molecular form of fibrin particularly resistant to break down by plasmin [31]. The mechanism by which peritonitis promotes progression to SP may start by the denudation of the mesothelium, which facilitates the peritoneal damage by the bioincompatible compounds from PD solutions, increases peritoneal permeability to glucose, and favours nonenzymatic glycosylation of submesothelial structural proteins and decrease in fibrinolytic capacity. Furthermore, peritonitis is associated with the increased intraperitoneal. Expression of TGF-*β* and other cytokines and growth factors that may accelerate the fibrotic process of the peritoneum [32].

(2) Time on PD: some authors [17–19], but not others [8, 9], have found a relationship between months on PD and the incidence of SPS. The main factor appears to be the prolonged exposure to glucose from PD solution [33], which is able to stimulate TGF-*β* and fibroblast growth factor (FGF) productions by MC [22, 34]. In addition, we have observed a correlation between the time on PD and the progression MMT [10].

(3) Poor biocompatibility of dialysis fluids: high glucose concentration, glucose degradation products (GDPs), advanced glycation end-products (AGEs), low pH, and lactate buffer in current PD solutions are all factors that have been implicated in peritoneal fibrosis [32, 35, 36]. These compounds have been associated to diminished production of phospholipids by MC, impaired phagocytosis capacity of macrophages, decreased activation of neutrophils [37] and lymphocytes [38], and direct toxicity of fibroblasts. Although the PD fluid components are risk factors for SP [37, 38], it is not always possible to identify the triggering agents for the progression of SP. Chlorhexidine and povidone iodine, employed to sterilize PD connections and preparation of surgeries, have been also implicated in the progression of SP [39, 40]. The peritoneal catheter as well as bags and tubes for dialysis are other risk factors that may cause reactive fibrosis [9]. Low pH, GDPs, and AGEs are shown to have a great capacity for stimulating the production of TGF-*β* [41].

(4) On the other hand, evidence for genetic predisposition to SPS has also been proposed. In fact the Japanese population in PD seems to be more prone to develop EPS [42]. Similar to other diseases, the genetic factors may predispose certain PD patients to develop EPS. In this case, the genetic polymorphisms of genes related to fibrosis and inflammatory processes may get a great importance in triggering the EPS. This is the case of single nucleotide polymorphisms (SNPs) in the promoter region of the interleukin-6 (IL-6), which has been related to transporter status [43]. However, there are few studies about the polymorphisms and their association with EPS, due to the large number of patients that are needed to do these studies and the low frequency of this pathology. In this regard, there is a study that associated RAGE polymorphisms with EPS; however, due to the low number of patients studied this relationship does not reach statistical significance [44]. 

There are a number of genes candidate to be studied which may have polymorphisms as toll-like receptors, inflammatory cytokines, chemokines, and several growth factors. Currently it is known around 2.4 million SNPs in the human genome and it is estimated that there may be millions more. In the future, it will be known which of these variants may be related with the development of EPS or in the perpetuation of SPS in EPS so that patients may have an individualized treatment trying to prevent its development. 

Table 1 summarizes the effects of TGF-*β* into the peritoneum. We have recently demonstrated that TGF-*β* is definitively one of the most important molecules in the initiation and perpetuation of peritoneal damage in PD. Experimentally, we used [14] strategies to identify the leading roles of TGF-*β* in peritoneal damage. First, in our mouse model we induced PD MMT, fibrosis, and type-I PM failure injecting daily glucose-rich PD liquid for 5 weeks. Two more groups received the same stimulus and a cotreatment with inhibitors of TGF-*β* two peptides (P17 and P144). Fibrosis, angiogenesis, and MMT decreased at the end of treatment also the PM function was preserved. Second, mice were infected with adenovirus encoding active TGF-*β* by intraperitoneal injection, and animals were killed on day 4 after infection. We reply all anatomical and functional changes induced by PD fluids but also find evidence of local endothelial cells (CD31+) conversion to fibroblasts, reinforcing the hypothesis that TGF-*β* and MMT are keys in the damage induced by PD liquids in PD.

### 2.3. From MMT to SPS

The importance of establishing a connection between MMT, SPS, and EPS is the potential therapeutic and preventive effect of blocking this axis. Also emerging evidence suggests that partial or total blockage of the MMT prevents early stages of PM fibrosis and angiogenesis and preserves the PM function [14]. Moreover, current studies show that TGF-*β* is probably the most important molecule in the PM failure development and so acts on a single molecule, the TGF-*β*, and facilitates therapeutic approach [14, 45]. In fact we have shown that blockade of TGF-*β* significantly attenuated PM failure induced by PD fluids [14].

**Figure 1:**
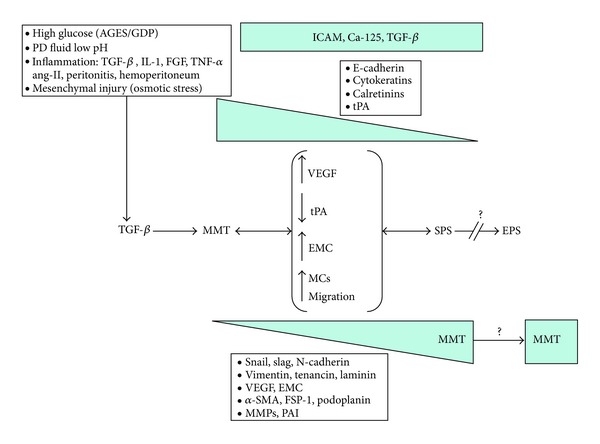
Mechanism for MMT, SPS, and EPS induction as a single process. The PD fluids bioincompatibility induces overproduction of TGF-*β* that initiates and perpetuates the MMT. MMT includes angiogenesis (VEGF), decreased in fibrinolytic capacity by decrease in tPA, increased in extracellular matrix component production (collagen-1, fibronectin, etc.), and migration mediated by MMPs. MCs lost their gene expression of E-cadherin, cytokeratins, and other and gain the expression of snail, slag, N-cadherin, and so forth. All of these changes become to peritoneal fibrosis and sclerotic peritoneal syndromes (SPS) which are originally reversible. MMT increases in parallel to fibrosis but its role in EPS pathogenesis is unknown. EPS is considered as irreversible process. ICAM, TGF-*β*, and Ca-125 expression remains stables.

One of the biggest problems to establish the definitive connection between SPS and EPS is that the EPS animal model has not been fully and properly developed. While in our mice PD model in 4 or more weeks reaches the typical changes induced by PD fluids on humans, the peritoneal fibrosis model with chlorhexidine results too much artificial and extremely aggressive for PM. The experimental development of an appropriate EPS model is mandatory. Possibly the most appropriate EPS mice model would be to maintain long term (months) in PD according to our model of SPS. Once accepted this limitation, the current data suggest that MMT and SPS are part of the process. We have analyzed serially PM pieces of mice in PD at baseline, 15, and 30 days and we found a linear correlation between time on PD, the thickness of the PM, and the number of MCs cytokeratin (+) and FSP-1 (+) in the submesothelial area (unpublished data by us). This phenomenon was accompanied by local progressive loss of the mesothelial monolayer which indicates an important participation of the MMT in the development of peritoneal fibrosis and MCs migration to submesothelial area (unpublished data by us). Using a TGF-*β* adenovirus model, we found early MMT at day 4 after stimuli intraperitoneal injection that was correlated with PM fibrosis [14]. Similar finding was found by others [45]. Clinically, in MCs serially isolated and cultured from PD effluents, the MMT was present progressively over time in PD and is associated with solute transport disorders and ultrafiltration failure [46]. In PM biopsies from 35 PD stable patients performed during the first 2 years on PD, we demonstrated that the first morphological change in peritoneum that appears as a consequence of PD is submesothelial thickening partially caused by the MMT. This phenotype change is associated with an increase in peritoneal solute transport independent of the number of capillaries present in the tissue [11]. Reached this point the following questions arise, could have peritoneal fibrosis without MMT? or more specifically, could have MMT without the participation of TGF-*β*? Experimental data by us [13, 14] and others [47] indicate that blocking MMT in different degrees result in a significantly attenuation of structural and functional changes of PM. Using the adenovirus (TGF-*β*) and our PD mice model, the double submesothelial staining for cytokeratin (+) and FSP1 (+) was positive in approximately 37% of activated fibroblasts, indicating its epithelial origin [14]. However, the peritoneal fibrosis is inhibited in more than 50% indicating that direct inhibition of TGF-*β* with anti-TGF-*β* peptides inhibited other effects of this molecule as the activation of regional fibroblasts. Promising results have been also obtained acting on immune system [48], on AGEs accumulation, or on renin-angiotensin system (ACE, AR-II, Paricalcitol) [49] and BMP-7 which also modulate directly or indirectly the TGF-*β* [13]. These arguments lead us to conclude that TGF-*β* is a key in the initiation and possibly perpetuation of an uncontrolled MMT, which leads to fibrosis and SPS (Figure 1).

### 2.4. From SPS to EPS

The next question is as follows: at which point the SPS becomes an irreversible process to become EPS? The “two-hit” hypothesis explains the EPS as the result of the PD injury. Two factors are required for the onset of EPS: a predisposing factor, such as peritoneal deterioration from persistent injury caused by peritoneal dialysis (the first “hit”), and an initiating factor, such as inflammatory stimuli superimposed on the chronically injured peritoneum (the second “hit”). Peritoneal deterioration (consisting of mesothelial denudation, interstitial fibrosis, vasculopathy, and angiogenesis) leads to an increased tendency toward plasma exudations that contain fibrin and coagulation factors. The fibrins in the exudates contribute to the intestinal adhesions and formation of fibrin capsule. Inflammatory stimuli caused by infectious peritonitis are superimposed on the damaged peritoneum and act as an initiating factor to trigger the onset of EPS. Inflammatory cytokines also induce activation and proliferation of the peritoneal fibroblasts, promoting peritoneal fibrosis and intestinal adhesions. The relationship between the extent of the first and second “hits” can be demonstrated. The extent of peritoneal damage (the first “hit”) increases with the duration of peritoneal dialysis. The onset of EPS depends on the total intensity of both lesions: peritoneal damage and inflammatory stimuli. For the onset of EPS, the total intensity must be greater than a given threshold. The extent of the inflammatory stimuli (the second “hit”) required for the onset of EPS therefore decreases as the duration of peritoneal dialysis increases [42, 50].

In both cases (acute and chronic peritoneal injury), the TGF-*β* is activated with subsequent initiation and perpetuation of MMT and its deleterious effects (fibrosis, angiogenesis, etc.). However, it is very difficult to establish the point of no return in peritoneal lesions clinically because patients with type-I PM failure usually recover functionality and possibly tissue damage with rest peritoneal [51]. In experimental animals, data about fibrosis reversibility are not available. Unfortunately, the initial degree of PM fibrosis has been determined in very few cases (peritoneal biopsies not available). Finally a genetic component cannot be ruled [43, 44]. 

### 2.5. From MMT to EPS

**Figure 2:**
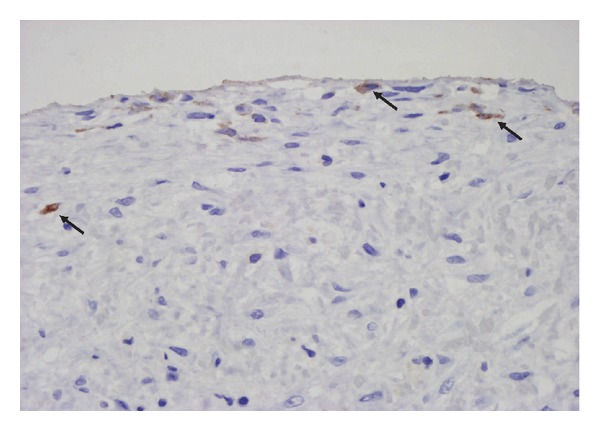
Evidence of MMT in EPS. Light microscopy analysis of a parietal peritoneal biopsy from a patient with EPS. Despite significant denudation of the peritoneal membrane, a submesothelial cytokeratin staining (brown) in submesothelial area is observed. This cytokeratin staining suggests the superficial precedence of these cells (arrows). Magnification ×200.

In both, experimental animals [14, 45] and human peritoneal biopsies from patients within 2 years in PD [11], it seems clear that MMT is an early phenomenon able to determine the degree of peritoneal fibrosis and the future of the PM. But no information about MMT in patients with long term in PD or diagnosis of EPS is available. It is possible that MMT may be an initial phenomenon and few signs of it are in severe stages of fibrosis (Figure 1). However, in bridles and postsurgical adhesions, we have found MMT signs (unpublished data by us), and Bowel adherences may represent an intermediate degree between the SPS and EPS (our unpublished data by us), which encourages to conduct studies aimed to find MMT peritoneum with EPS. These findings represent important evidence linking both processes, but indirect evidence may also be marked. In human studies [10] and in experimental animals (unpublished data), our studies demonstrated a direct relationship between MMT and time on PD. Similarly, the several studies showed a parallel between EPS and time on PD [52, 53]. Another important fact is that peritoneal function studies also show a parallel between high frequency of MMT of MCs, high Cr-MTC, and low ultrafiltration. Indeed we observed a higher frequency of mesothelial fibroblastoid phenotype in patients with type Cr-MTC >11 mL/min [54]. Furthermore, as is well known, patients with EPS even displayed these with SPS showed similar functional PM deterioration [9, 55, 56]. Another indirect association between these two events is peritonitis. Yáñez-Mo and coworkers [10] found that the frequency of nonepithelioid MC was associated with episodes of peritonitis, this means that peritonitis leads to the MMT. In the case of the EPS, there are some studies in the literature that correlate it with peritonitis events. Previous studies suggest that peritonitis may predispose to EPS, especially if this is caused by *Staphylococcus aureus*, fungi, and/or *Pseudomonas* [9, 57]. There is also an association between persistent infections such as tuberculosis peritonitis and EPS [58]. Although peritonitis and EPS are highly associated in several studies it is also known that, especially in a long-term case, EPS may occur without peritonitis. Moreover, patients that have suffered from more events of peritonitis have a higher incidence of MMT and EPS, which suggest again that these processes are related. Finally, we have analyzed more than 10 peritoneal biopsies from patients with EPS where we had found a significant amount of mesothelial cells (CK +) in the peritoneal submesothelial area, which indicates that despite the significant denudation of the peritoneal MCs monolayer persists and important migration of MCs to compact zone (Figure 2). 

## 3. Conclusion

TGF-*β* appears to be the most important molecule in the initiation of MMT and peritoneal fibrosis. MMT is present from early stages of peritoneal fibrosis and is perpetuated over time. Current data support a connection between MMT and SPS. However, the jump from SPS to EPS and the connection between MMT and EPS have not been fully established. We concluded that the MMT can be a therapeutic target, the blockade of which could be a benefit especially in initial stages of the process.

## Figures and Tables

**Table 1:** Implication of TGF-*β* in peritoneal fibrosis.

(i) Activates quiescent fibroblasts into myofibroblasts
(ii) Increases fibronectin production by fibroblast and MC
(iii) Induces the expression of connective tissue growth factor (CTGF) by MC
(iv) Stimulates the synthesis of PAI, the natural inhibitor of tPA, contributing to the generation of an antifibrinolytic environment
(v) Increases matrix synthesis and inhibits matrix degradation by decreasing the ratio MMPs/TIMPs
(vi) Induces MMT of MC
